# Hydration and strength development in blended cement with ultrafine granulated copper slag

**DOI:** 10.1371/journal.pone.0215677

**Published:** 2019-04-26

**Authors:** Yan Feng, Qinli Zhang, Qiusong Chen, Daolin Wang, Hongquan Guo, Lang Liu, Qixing Yang

**Affiliations:** 1 School of Resource and Safety Engineering, Central South University, Changsha, Hunan, China; 2 Energy School, Xi’an University of Science and Technology, Xi’an, Shanxi, China; Chiang Mai University, THAILAND

## Abstract

This study aims at evaluating the effect of ultrafine granulated copper slag (UGCS) on hydration development of blended cement and mechanical properties of mortars. The UGCS with the median particle size of 4.78 μm and BET surface area of 1.31 m^2^/g was used as a cement replacement to prepare blended cements. Hydration heat emission of blended cement and mechanical performance of mortars were investigated by using isothermal calorimetry and strength tests, respectively. X-ray diffraction (XRD), thermogravimetric analysis (TGA), and scanning electron microscopy (SEM) were applied to the analysis of pozzolanic reaction and hydration products. The results illustrate that UGCS has influence on the hydration heat evolution of blended cement due to its filler effect and pozzolanic reaction. The cumulative hydration heat of blended cement is reduced by partial cement replacement with UGCS. The test mortar prepared by using blended cements with 30 wt. % UGCS shows a retardation of strength development with a low value at early ages (7 days) and a rapid growth at later ages (28 days). The 90-day compressive strength of test mortar is 45.0 MPa close to that of the control mortar (49.5 MPa). The obtained results from XRD and TGA analysis exhibit an increase in calcium hydroxide (CH) consumption and calcium silicate hydrates (C–S–H) formation in blended cement pastes with curing time. The cement replacement with UGCS induces changes in microstructure of blended cement paste and chemical composition of hydration products.

## Introduction

Recently, the utilization of industrial by-products as supplementary cementitious materials (SCMs) in cement and concrete has attracted considerable attention for the technological, economic, and environmental benefits [1–4]. Copper slag is a by-product generated from the process of copper manufacturing [5]. Granulated copper slag (GCS) is an amorphous material due to rapid water cooling, in which the glassy phase consists mainly of ferrous oxide (FeO), silicon dioxide (SiO_2_), and minor amount of other compounds [6]. The high content of reactive SiO_2_ in GCS endorse its latent pozzolanic properties [7]. Nevertheless, the low activity of GCS adversely affects the mechanical performance of cement and concrete at early ages when used as partial substitution of cement [8,9].

Researches [10,11] have demonstrated that pozzolanic activity of glassy materials is mainly depended on their chemical composition, fineness, and glassy phase content. Accordingly, auxiliary activation techniques, such as thermal, chemical, and mechanical activation, have been used for enhancing the reactivity of pozzolanic materials [12]. Mechanical activation is able to induce the modifications in specific surface area, surface structural, and other defects [13–15]. When used as cement replacement, finer materials affect the mechanical performance of cement products due to both filler (physical) and pozzolanic (chemical) effects [16–19]. The filler effect is related to the packing characteristics of fine particles which can fill the viods of hydration products. This effect will contribute to the improvement of mechanical strength without chemical reaction. The pozzolanic effect is due to the capability of providing reactive SiO_2_ that reacts with calcium hydroxide (CH) generated during cement hydration. However, materials with conventional grinding are difficult to achieve high fineness for maximizing their activities and replacement levels. Bouaziz et al. [20] investigated the effect of conventional milling on the fineness of granulated blast furnace slag (GBFS). They found that the fineness of GBFS does not exceed the specific surface area of 0.4 m^2^/g after 10 h of conventional milling. As one of the efficient ways, mechanical activation with high-energy milling has been successfully applied to other materials such as GBFS and fly ash (FA) [13,14]. Kumar et al. [13] investigated the effect of mechanically activated GBFS with an attrition mill on the early strength development of blended cement pastes. In another study, the authors [21] reported the effect of mechanical activation on the reactivity of FA when used for geopolymer synthesis. They found that high-energy milling is efficient for materials to achieve high fineness within 3 h of milling, but is limited by the particles agglomeration, with maximums of 1.8 and 2.0 m^2^/g for GBFS and FA, respectively. Their studies show that the ultrafine materials exhibit higher reactivity, and the compressive strength development of blended cement pastes is mainly dependent on the fineness instead of milling duration. Previous studies investigating utilization of copper slag in cement and concrete focus on the materials with fineness ranging from 0.3–0.7 m^2^/g [8,22,23], which is a big obstacle to its wide application.

In the present study, ultrafine GCS (UGCS) was obtained by using vibratory milling. The hydration heat evolution of blended cement was detected by isothermal calorimetry. The effect of UGCS on the compressive strength development of mortars was investigated. X-ray diffraction (XRD), thermogravimetric analysis (TGA), and scanning electron microscopy (SEM) were used to analyze the pozzolanic reaction and hydration products.

## Materials and methods

Portland limestone cement (PLC) used in the present study was CEM Ⅱ/A-LL 42.5 R and copper slag was provided by a copper smelter via water granulation of liquid slag from the settling furnace. The as-received slag was screened by standard sieves and the feedstock particles with fineness below 150 μm in diameter were obtained. High-energy milling test was carried out in a vibrating mill (Humboldt, Germany) with a vibrating amplitude of 10 mm and a frequency 1000 rpm. The milling was performed using cylpebs with a diameter of 12 mm for duration of 3 h and the UGCS in this study was obtained.

The chemical compositions of UGCS and cement were performed using X-ray fluorescence spectroscopy (XRF). The UGCS samples were sent to Changsha Research Institute of Mining and Metallurgy co, .Ltd for determining content of iron with different valence. The amorphous phase was determined by using XRD (Empyrean, PANalytical) and SEM (Zeiss Merlin) equipped with energy dispersive X-ray spectroscopy (EDS). The XRD analysis was performed with Cu Kα radiation operating at 45 kV and 40 mA. The scanning range was from 10 to 70° with a step size of 0.026. SEM/EDS was performed with an accelerating voltage of 20 kV and an emission current of 1.0 nA [24]. The particle size distribution within the range of 0.04–500 μm was determined by laser diffraction using a particle size analyzer (CILAS 1064). The BET surface area was measured using a Micromeritics Flowsorb II 2300.

Blended cements were prepared by using UGCS as cement replacement with substitution ratios of 30 wt. % and 50 wt. %. PLC served as the reference cement. The hydration heat at early ages was continuously monitored by an isothermal calorimeter (TAM Air device, TA Instruments) at 25°C within 72 h. The analysis was performed on blended cements and PLC with a water-to-binder ratio of 0.5 in accordance with Chinese national standard GB/T 12957–2005 and literature [8]. Based on the results, blended cement pastes with 30% UGCS and PLC were cured at 20 ± 2°C and > 95% humidity for 7, 28, and 90 days. After curing, the samples were dried in an oven at 50°C for 3 days to stop hydration and then stored in a desiccator for further analysis and tests.

Control and test mortars were prepared using PLC and the blended cement with 30% UGCS as binders, respectively. The strength tests were carried out according to Chinese standard GB/T17671-1999. Each of the binders was mixed with water and CEN standard sand with water to binder ratio of 0.5 and cement to sand ratio of 1:3. These mixtures were cast into 40×40×160 mm steel molds and compacted on a vibration table for 1 min. All the mortar samples were demolded after 24 h and stored under laboratory conditions (20 ± 2°C and > 95% humidity). Compressive strength of mortars was examined at the curing age of 7, 28, and 90 days [25,26]. For each measurement, three specimens were used and the average values was reported as the result.

The bulk paste samples of blended cements at different curing ages were crushed to powder. In order to determine the hydration products of blended cement, the mineralogy was determined by using XRD in the scanning range from 10° to 70° (conditions and analysis are described above). Qualitative phase analysis was performed by HighScore software package (PANalytical, version 4.7).

The paste samples of blended cements and PLC were subjected to simultaneous thermal analysis to further determine the hydration products and progress of blended cement. A STA 409 instrument (Netzsch, Germany) with thermogravimetric analysis (TGA) and differential thermal analysis (DTA) was used to quantitatively estimate the thermal decomposition of hydrate phase. The tests were carried out under nitrogen atmosphere with a flow rate of 100 ml/min.

Chemical composition and microstructure of the hydration products were analyzed by using SEM (Zeiss Merlin) equipped with energy dispersive X-ray spectroscopy (EDS), the conditions and analysis are described above. Analysis was performed on carbon-coated polished surfaces.

## Results and discussion

### Characterization of UGCS

**Fig 1:**
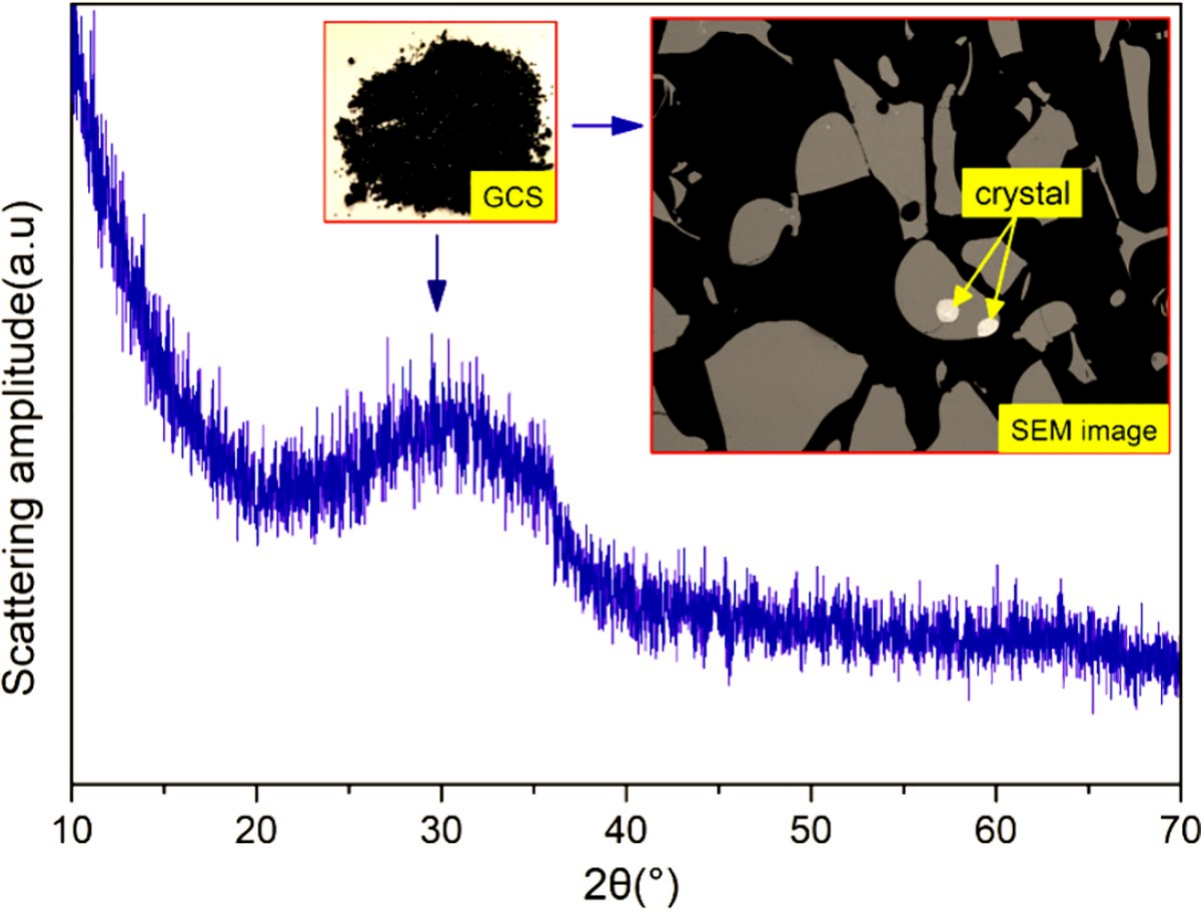
XRD pattern of UGCS and SEM image of granular GCS.

The chemical composition of the UGCS and PLC is shown in Table 1. The main oxides of UGCS are SiO_2_, FeO, and Fe_2_O_3_ accounting for 77% of the total mass, while Al_2_O_3_ and CaO are present in minor amounts. Fig 1 shows the XRD pattern of UGCS, which reveals amorphous characteristics as reflected by a broad diffuse band located between 20° and 40° without obvious diffraction peaks. Besides, the glassy particles are observed in SEM micrographs (Fig 1) to possess homogeneous structure with clean and smooth surface. Only a few crystalline phases in form of inclusions are identified to be magnetite (Fe_3_O_4_) and chalcopyrite (CuFeS_2_) according to the EDS results, which are inhomogeneously distributed over the particles. These results indicates that the UGCS sample consists predominantly of glassy phases, in which the reactive constituents, i.e., SiO_2_ and Al_2_O_3_, can potentially take part in pozzolanic reaction, with the content of 40% higher than the minimum requirement (25%) in accordance with BS EN 197–1 (2011).

**Table 1:** Chemical composition (%) of UGCS and PLC.

Material	SiO_2_	FeO	Fe_2_O_3_	Al_2_O_3_	CaO	MgO	Zn	Cu	K_2_O	SO_3_	Others
UGCS	36.00	27.01	13.76	4.10	3.60	1.51	1.10	0.81	–	–	12.11
PLC	18.10	–	2.80	4.90	62.10	1.20	–	–	1.20	3.70	6.00

**Fig 2:**
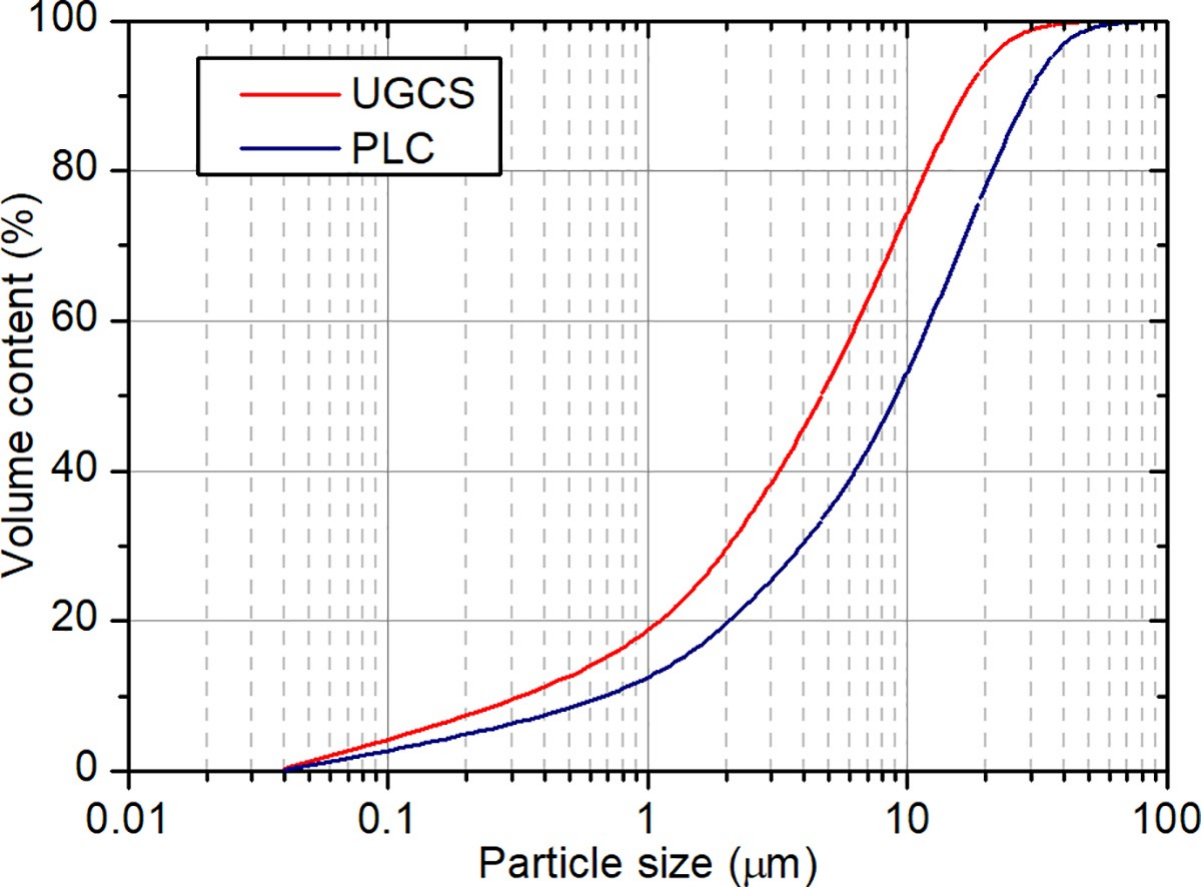
Particle size cumulative distribution of UGCS and PLC.

Fig 2 shows the cumulative particle size distribution of UGCS and PLC. The characteristic particle sizes and specific surface area are presented in Table 2. With the granulometry of 90 vol% below a size of 15 μm, UGCS has a narrower and finer particle sizes distribution when compared with PC. By providing more nucleation sites during tricalcium silicate (C_3_S) dissolution, the ultrafine particles in UGCS can accelerate the precipitation and growth of calcium silicate hydrates (C–S–H) [27,28]. This will consequently accelerate the early hydration process of cement. The UGCS studied here with BET surface area of 1.31 m^2^/g is much finer particle size than that reported in other investigations with the fineness between 0.3–0.7 m^2^/g [8,22,23], which increases the surface available for pozzolanic reaction between UGCS particles and CH.

**Table 2:** Characteristic particle sizes and specific surface area of UGCS and PLC.

Material	D10	D50	D90	Specific surface area (m^2^/g)
PLC	1.01	9.56	26.55	0.47
UGCS	0.65	4.78	14.84	1.31

### Isothermal calorimetry

**Fig 3:**
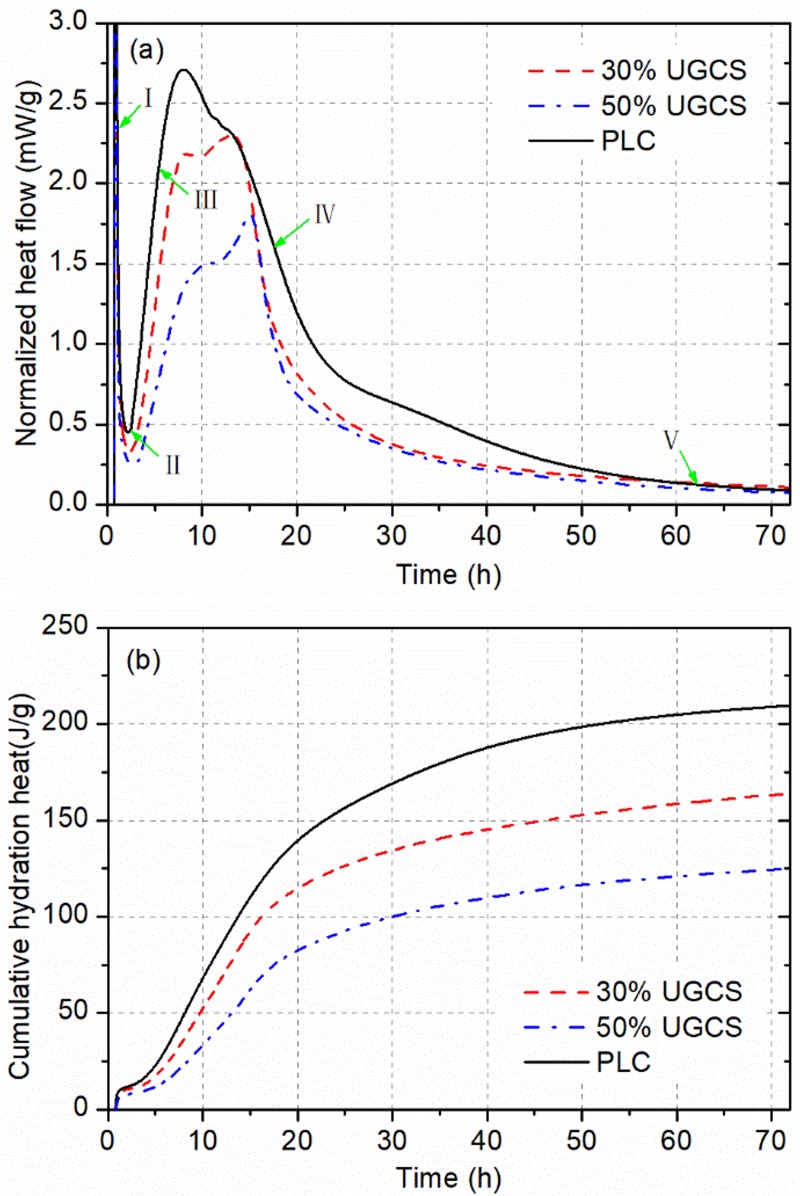
Isothermal calorimetry results for blended cements and PLC at 25°C: (a) normalized heat flow and (b) cumulative hydration heat.

Fig 3 shows the normalized heat flow and cumulative heat of blended cements with UGCS at 25°C. The profile of PLC is given as a reference. Characteristic values of hydration heat evolution are summarized in Table 3.

**Table 3:** Characteristic values of hydration heat evolution for blended cements and PLC.

Sample	The induction period (h)	Peak value (mW/g)			Total heat emission (J/g)			
		The first peak	The second peak	The third peak	12 h	24 h	48 h	72 h
UGCS (50)	2.7–3.4	15.2	1.5	1.8	44.2	90.8	115.3	124.8
UGCS (30)	2.5–2.9	12.4	2.2	2.3	68.5	124.4	151.5	163.9
PLC	2.2–2.5	17.7	2.7	–	85.8	153.6	196.8	209.6

As described in [29,30], there are five stages of hydration heat evolution for PLC: the initial period (I), the induction period (II), the acceleration period (III), the deceleration period (IV), and the period of slow continued reaction (V). A strong exothermic signal is recorded immediately after mixing cement with water in the initial period (I) to yield the first peak in Fig 3(A), which is attributed mainly to the wetting of cement powders and rapid dissolution of C_3_S [31]. The dilution effect of UGCS that affects the hydration heat evolution of cement over the early ages, induces a reduction in the peak value of blended cement, as shown in Table 3. Based on the hypothesis of superficially hydroxylated layer, the subsequent sharp decrease in exothermic rate is related to the superficial hydroxylation of C_3_S surface reducing its apparent solubility. This is followed by the induction period (II) with a low rate of heat release, during which a dynamic equilibrium is established between the dissolution of C_3_S and the early growth of C–S–H. The delay in achieving the equilibrium for blended cement due to the cement dilution is exacerbated by increasing the addition of UGCS. With partial replacement of cement by UGCS, the water-to-cement ratio was increased resulting in the prolonged duration for pore solution to reach a supersaturation with respected to C–S–H. The extension of the induction period can be also explained by the dilution effect of UGCS. Subsequently the acceleration period (III) comes with the formation of second exothermic peak. During this period, the hydration of cement is dominated by the rapid and massive nucleation and growth of the C–S–H and CH along with a significant increase in the rate of heat release. The rate for PLC achieves a maximum at the end of the acceleration period (8.0 h). With increasing the dosage of UGCS in blended cement, the second peak becomes weak and nearly disappear. Nevertheless, the blended cements show reductions in the peak value lower than the ratios of cement replacement, which is attributed to the physical (filler) effect of UGCS [32]. The UGCS with high fineness can provide more nucleation sites for the precipitation and growth of C–S–H, accelerating the hydration heat release of C_3_S. The strong exothermic signal is followed by the deceleration period (IV) with a sharp decrease in the heat release rate owing to the transition from chemical controlled process to diffusion controlled process for cement hydration [28]. Unlike PLC, which demonstrates a continuous decrease in the heat release rate during this period, a new exothermic peak (the third peak) is formed mainly due to the chemical effect of UGCS [32]. The chemical effect is ascribed to the pozzolanic reaction, in which UGCS reacts with CH generated by the cement hydration to form C–S–H. With the formation and growth of thick hydrated layer on the surface of C_3_S particles, the diffusion of reactants becomes slower [31]. During the slow continuing reaction period (V), the hydration finally reaches a very low rate and develops continuously with a low heat output. The chemical effect of UGCS is also confirmed by the increased exothermic rate for blended cement during the period V, as shown in Fig 3(A), as the cement replacement ratio decreases. Due to the low reactivity of UGCS, the pozzolanic reaction comes into play in the cement hydration after the acceleration period, during which a large amount of CH is generated to provide alkaline environment for the decomposition of UGCS and reactants for pozzolanic reaction.

The cumulative heat curve and total heat emission at different time are presented in Fig 3(B) and Table 3, respectively. As shown in Fig 3(B), the hydration heat for blended cement accumulates in a similar way to that for PLC with the rapid increase in cumulative heat within the first 30 h and then tending to level off. This indicates that the hydration process of blended cements were dominated by the cement hydration in the early period. As shown in Table 3, the blended cements exhibit a significant decrease in the cumulative heat especially at 12 h owing to the cement dilution effect. The more UGCS was incorporated in cement, the more reduction in the total heat emission was caused. However, the total heat emission of blended cements exceed that released independently by the hydration of cement in these binders due to the chemical effect of UGCS. This is consistent with the results from heat flow as described above. Another conceivable explanation was reported by Han et al. [33], who proposed that the consumption of CH by pozzolanic reaction in turn promotes the cement hydration. These results indicate that UGCS exhibits pozzolanic activity, which affects the heat evolution of cement hydration. The significant decrease in the cumulative hydration heat for blended cement is beneficial in reducing autogenous shrinkage of cement and concrete products.

### Compressive strength

The compressive strength and strength development rate (SDR) for the mortars prepared by using PLC and blended cements with 30% UGCS (control mortars and test mortars) are shown in Table 4. A retardation of strength development can be seen for the test mortars when compared with the control mortars. As shown in Table 4, the test mortar gains compressive strength of 26.7 MPa at 7 d, which is less than 60% of the strength gained by the control mortar. The negative effect on the early-age strength due to the cement dilution can be mitigated by prolonging the curing time [34,35]. The compressive strengths for test mortars at 28 d and 90 d are acquired as 87% and 91% of that for the control mortars, respectively. Although the test mortar show low strength values at early ages, its strength gain rates are much higher than that for the control mortar especially in the curing period of 7–28 d. This is attributed to the combined effect of cement hydration and pozzolanic reaction. Due to the low reactivity of UGCS, the pozzolanic reaction at early ages plays a secondary role in strength development, which is insufficient to compensate for the dilution effect. The strength development is gradually dominated by the pozzolanic reaction with curing time, resulting in a sharp increase in the strength value at later ages. The compressive strength of test mortar reaches a maximum of 45.0 MPa, which is close to the value (49.5 MPa) obtained by the control mortar. As shown in Table 4, the SDR of mortars is significantly affected by the curing time and UGCS. The control mortar shows a decrease in SDR from the period of 7–28 d to 28–90 d. This is related to the decrease in the un-hydrated cement particles. In the period of 7–28 d, the SDR value of test mortars is much higher than that of the control mortar associated with the pozzolanic reaction, which occurs between UGCS and CH with the formation of additional C–S–H gel. This also indicates that the effect of pozzolanic reaction on the strength development is more efficient in the period of 7–28 d. This effect is greatly weakened with curing time, but still exists in the period of 28–90 d. This demonstrates that the pozzolanic reaction of UGCS is a long-term process with contribution to the growth in compressive strength of test mortars even after 90 days of curing time.

**Table 4:** Compressive strength and strength development rate of mortars.

Sample	Compressive strength (MPa)			Strength development rate (MPa/d)	
	7 d	28 d	90 d	from 7 to 28 d	from 28 to 90 d
PLC	46.8	48.9	49.5	0.10	0.01
CS3	26.7	42.6	45.0	0.76	0.04

### Mineralogical analysis

**Fig 4:**
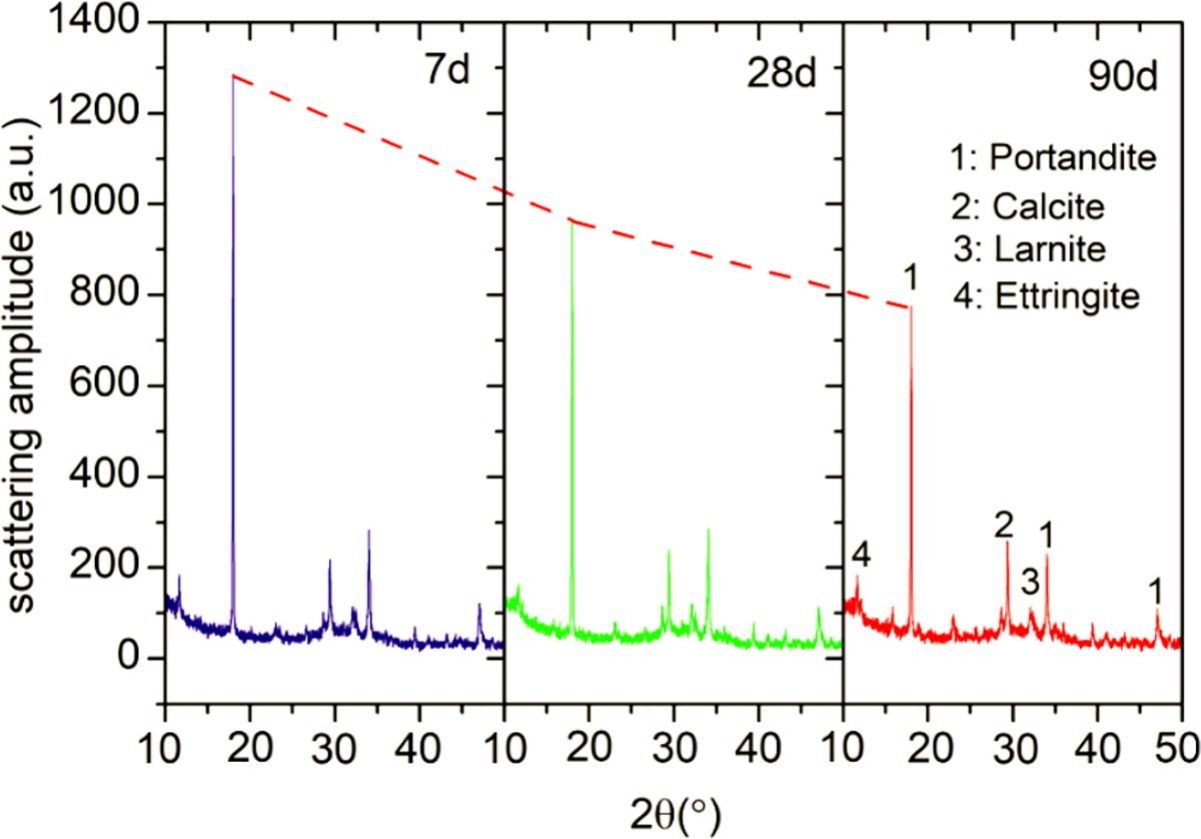
XRD patterns of the blended cement pastes with 30% UGCS at 7, 28, and 90 d.

Fig 4 presents the XRD patterns of the blended cement pastes with 30% UGCS after 7, 28, and 90 days of curing. The spectra in the region of 2θ between 10° and 50° are stacked to investigate the change in hydration products of blended cement with curing time. The blended cements at different ages show similar collections of diffraction peaks. It can be observed that the main minerals in the paste samples are portlandite (CH), calcite (CaCO_3_), larnite (β-C_2_S), and ettringite (AFt) [36]. Portlandite is generated during the hydration of cement with the presence as the primary crystalline mineral in the pastes. The calcite and larnite are also detected by XRD with lower intensities. The existence of calcite is due to the extender in PLC and the carbonation of CH during setting and hardening of the pastes [37]. As for β-C_2_S, it is present as unhydrated cement component owing to its low reactivity [38]. In addition, C–S–H gel generally identified as an amorphous phase is difficult to be detected by XRD.

It is well known that the amorphous silica, which is the reactive component in pozzolanic materials, can react with CH to produce additional hydration product. The degree of pozzolanic reaction is generally monitored by the consumption of CH in cement-SCM pastes. In our case, the diffraction peaks of CH in blended cement pastes appear at 2θ = 18.02° and 34.05°. The peak intensity of CH at 18.02° has been compared as shown in Fig 4. The results show a gradual and prominent decrease in this peak intensity with curing time, but with a residual portion remaining in the pastes at 90 d. This indicates that the incorporation of UGCS yields the pozzolanic reaction, which would proceed even after 90 days of curing time. In order to accurately determine the degree of pozzolanic reaction, quantitative analysis will be performed with taking the effect of CaCO_3_ into consideration in the following section [37].

### Thermal analysis

**Fig 5:**
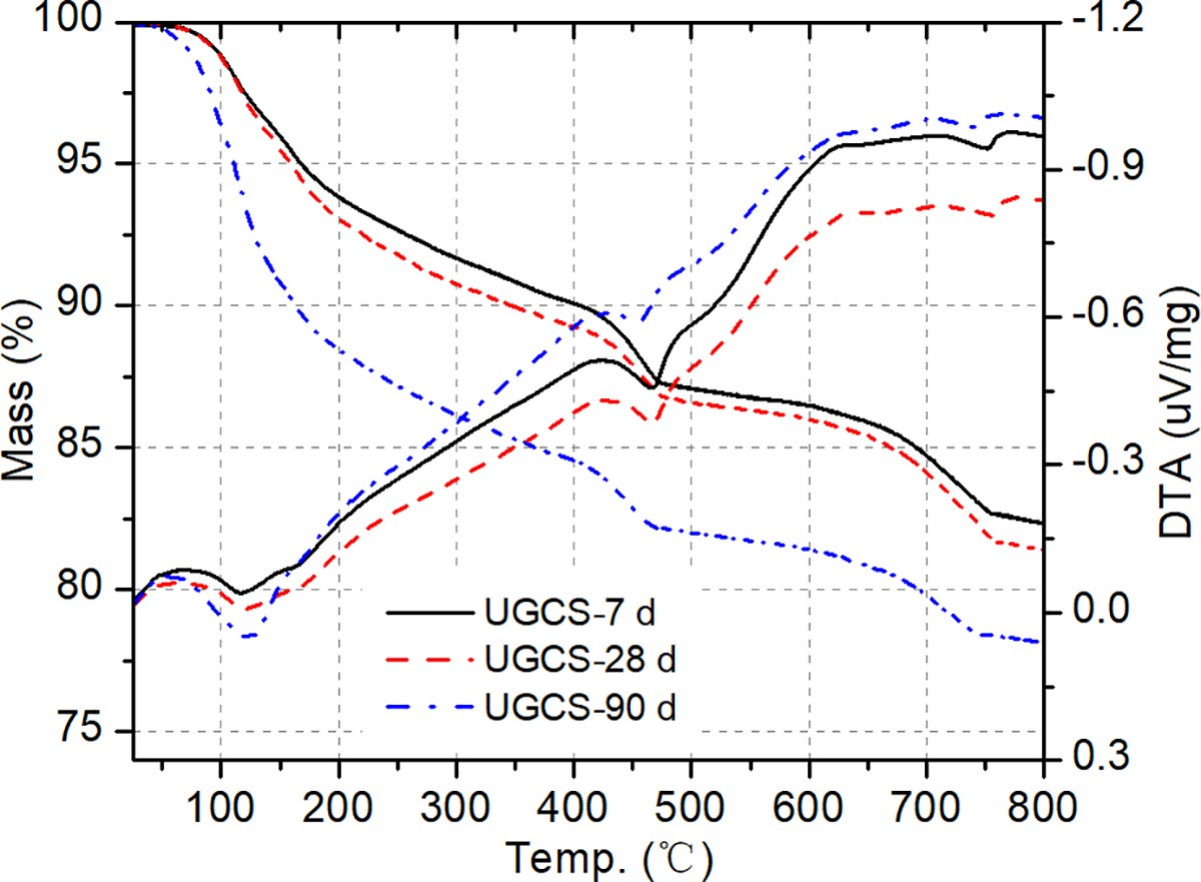
DTG/TGA curves of blended cement pastes at 7, 28, and 90d.

**Fig 6:**
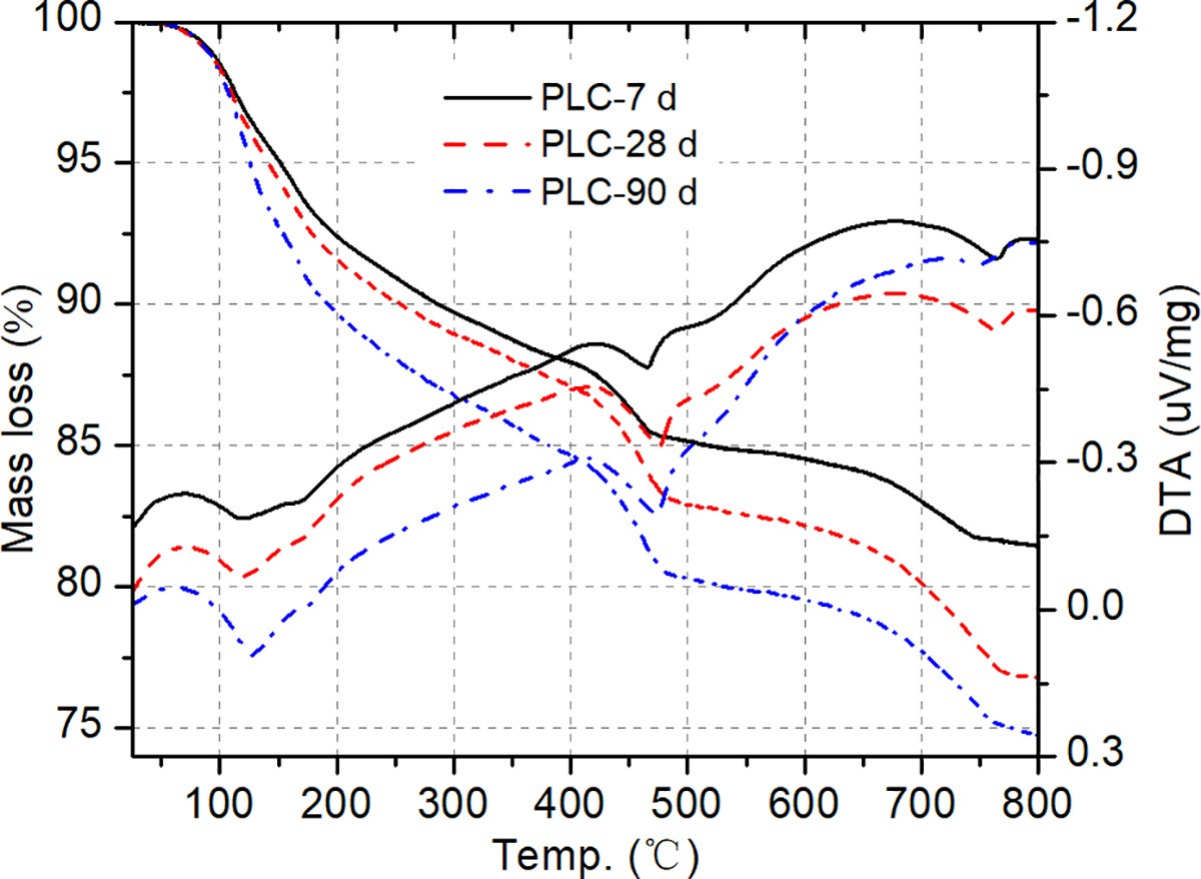
DTG/TGA curves of PLC pastes at 7, 28, and 90d.

TGA is generally used to identify cement hydration products by the weight loss within specific temperature ranges. The decompositions of hydrated and carbonated phases can be represented by the corresponding peaks occurring in the differential thermal analysis (DTA) curve. TGA/DTA curves of blended cement with 30% UGCS and PLC pastes are presented in Figs 5 and 6, respectively, after 7, 28, and 90 days of curing. DTA curves show three endothermic peaks in the particular regions. The first endothermic peak bounded between 70°C and 210°C is mainly due to the dehydration of C–S–H and decomposition of AFt [39], which is based on the identified minerals from the XRD analysis. The second peak in the range from 425–530°C is the characteristic of crystalized water released by the dehydroxylation of CH [40]. The last endothermic peak between 710 and 780°C corresponds the decarbonation of CaCO_3_ [41]. It is noticeable that no obvious evidence for the existence of monosulphate (AFm) can be found from both XRD and DTG analysis, which is in agreement with observations in other studies on the blended cement with CaCO_3_ or GBFS [41,42].

In order to evaluate the degree of pozzolanic reaction, the mass loss is employed to determine the quantity of CH consumption in the blended cement pastes [43]. With taking the carbonized part of CH into consideration, the total CH contents of blended cement and PLC pastes with curing time at 7, 28, and 90 d are presented in Table 5. The CH contents of PLC pastes at 7, 28, and 90 d are 14.13%, 14.89% and 15.87%, respectively. The increase in CH content of the PLC paste with curing time is due to the continuous hydration reaction. With a replacement of cement by 30% UGCS, the blended cement shows a decrease in the CH contents at different curing ages. This is attributed to the CH consumption by UGCS during the pozzolanic reaction. The percentages of fixed lime is an indicator of the capability for pozzolanic materials. The method described in literature [44] is employed to calculate the percentages of fixed lime for the blended cement paste and the results are shown in Table 5. The low percentage of fixed lime for the blended cement paste at 7 d indicates a low pozzolanic activity of UGCS at early ages, which is in agreement with the results reported by Rojas et al. [8]. With the development of pozzolanic reaction, more available lime is fixed and the value reaches a maximum of 28.80% after 90 days of curing. The fixed lime increases in the period of 7–28 d at a higher rate than that in the period of 28–90 d. The deceleration is consistent with the trend of strength development rate with curing time, which can be explained by the change of reaction environment. CH generated by cement hydration is consumed during the dissolution of UGCS particles and pozzolanic reaction. The CH consumption induces a reduction in pH value of pore solution in the pastes, slowing down the pozzolanic reaction. In comparison to the results reported in [45], the blended cements with 30% fly ash show similar percentages of fixed lime to that with 30% UGCS in our study at the same curing ages. This indicates that UGCS exhibits pozzolanic activity equivalent to fly ash. Besides, the results of TGA and strength test show a low percentage of fixed lime for blended cement and a high strength value for mortar at 90 d. This illustrates that UGCS has influence on the development of compressive strength not only by the pozzolanic reaction, but also by the packing effect of UGCS [46].

**Table 5:** CH contents and percentages of fixed lime for the blended cement pastes at 7, 28 and 90 d.

Sample	CH content (%)		
	7 d	28 d	90 d
PLC	14.13	14.89	15.87
Blended cement	6.93	6.32	4.58
Fixed lime (%)	14.67	19.29	28.80

### Microstructural analysis

**Fig 7:**
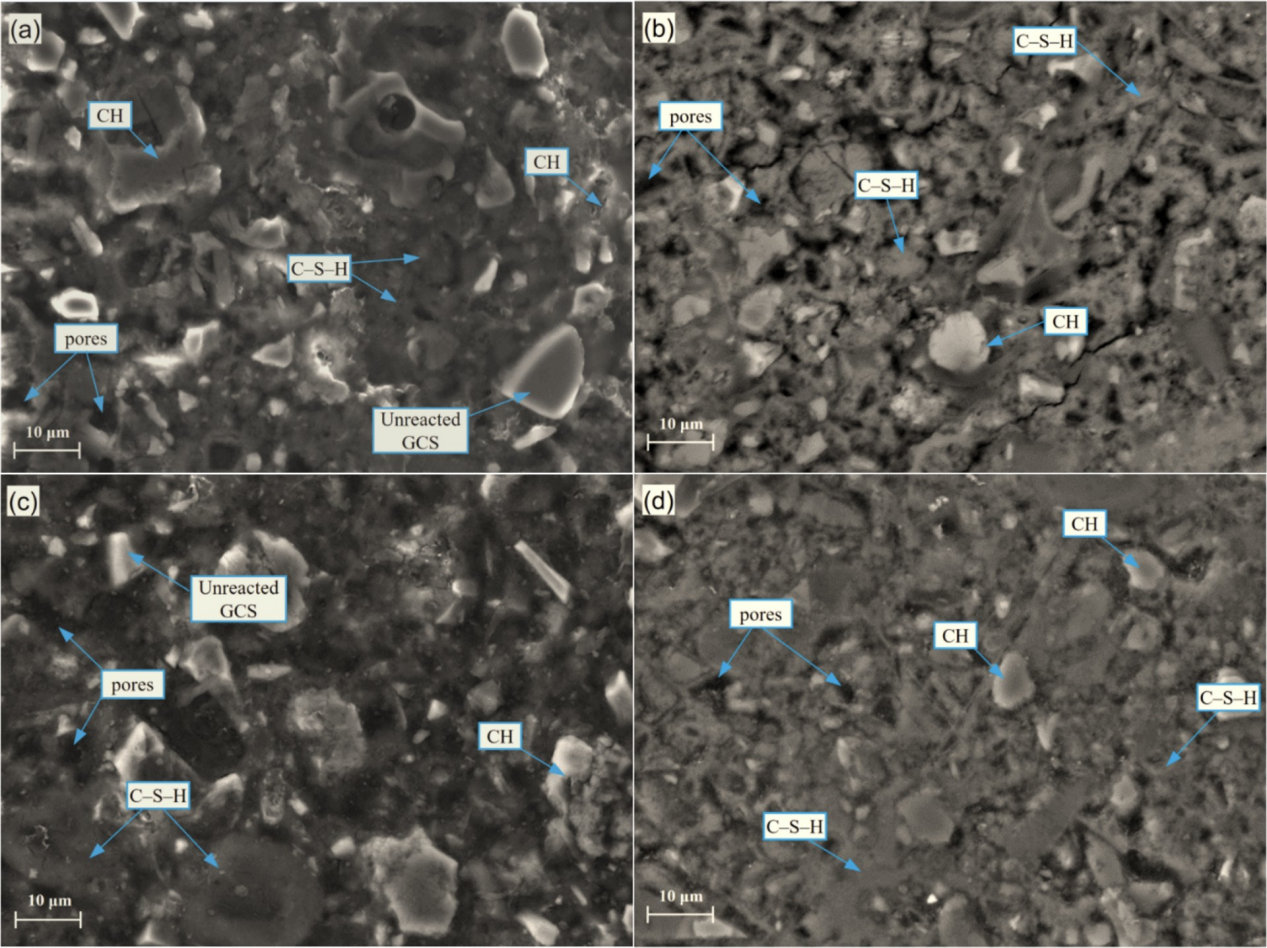
SEM images of blended cement and PLC pastes after 28 and 90 days of curing: (a) UGCS-28 d; (b) PLC-28 d; (c) UGCS-90 d; (d) PLC-90 d.

SEM micrographs of blended cement and PLC pastes at 28 and 90 d are shown in Fig 7. In the micrographs of PLC pastes (Fig 7(B) and 7(D)), mixtures have been observed with well crystallized CH, poorly crystallized C–S–H gel, AFt, and pores occupying most of the available volume in cementitious matrix and forming a compact microstructure. With partial replacement of cement by UGCS, as shown in Fig 7(A) and 7(C), the pastes show more loose and inhomogeneous microstructure with the presence of less C–S–H gel and more pores. This is consistent with the results from the XRD and thermal analysis and can be used to explain the decrease in compressive strength of test mortars. With development of cement hydration and pozzolanic reaction, the microstructure of blended cement becomes denser with the formation of more C–S–H gel. Moreover, only a few CH with irregular shapes have been detected in the blended cement pastes instead of the CH crystal with hexagonal plate structure as present in PLC pastes. This is attributed to the consumption of CH by UGCS during the pozzolanic reaction. Nevertheless, there are some unreacted UGCS in large particles remain embedded in the blended cement pastes even after 90 days of curing. A longer curing duration is required to fully develop the pozzolanic reaction, which is consistent with the results from thermal analysis.

EDS analysis was performed on paste samples to determine the effect of UGCS on the composition of hydration products. The average values of Ca/Si and Fe/Si atomic ratios for C–S–H gel are summarized in Table 6. Five different target areas in each sample were acquired for analysis. The Ca/Si ratios for PLC pastes after 28 and 90 days of curing are 2.23 and 2.47, respectively, with low concentration of iron. The replacement of cement with UGCS results in a decrease of Ca/Si ratio, which is attributed to the formation of additional C–S–H gel with low concentration of calcium during the pozzolanic reaction. The pozzolanic reaction develops accompanied by the growth of C–S–H gel of this type grows, diluting the concentration of calcium in C–S–H gel formed by cement hydration. The average value of Ca/Si ratio for blended cement paste decreases to 1.97 after 90 days of curing. Besides, the values of Fe/Si ratio of C–S–H gel in the blended cement paste is much higher than that in the PLC paste at certain ages (28 and 90 d). This change can be attributed to the chemical transformation of iron during the dissolution of UGCS and the subsequent pozzolanic reaction. The iron ions are released from the dissolution of UGCS particles as reactive components and then taking part in the pozzolanic reaction to form the C–S–H gel. This can be also confirmed by the increase in Fe/Si ratio for blended cement with curing time.

**Table 6:** Ca/Si and Fe/Si atomic ratios of C–S–H gel formed in PLC and blended cement pastes.

Atomic ratio	PLC (28 d)	PLC (90 d)	UGCS (28 d)	UGCS (90 d)
Ca/Si	2.23	2.47	2.10	1.97
Fe/Si	0.03	0.07	0.19	0.43

## Conclusion

Hydration and strength development in blended cement with ultrafine granulated copper slag (UGCS) were investigated by using isothermal calorimetry and compressive tests. XRD, DTA/TGA, and SEM were applied to the analysis of pozzolanic reaction and hydration products. The conclusions can be summarized as follows:

1. The UGCS is sufficiently obtained by using vibratory milling with median particle size of 4.78 μm and BET surface area of 1.31 m^2^/g. The high content of reactive constituents and amorphous phase in UGCS endorse its latent pozzolanic properties.
2. The cumulative hydration heat of blended cement is reduced by using UGCS as a cement replacement. The hydration heat evolution is significantely affected by cement replacement ratio at different stages.
3. The test mortar prepared by using blended cements with 30 wt. % UGCS shows a retardation of compressive strength development, with a low strength value at early ages (7 days) and a rapid growth at later ages (28 days). The 90-day compressive strength of test mortar is 45.0 MPa close to that of the control mortar (49.5 MPa). It is possible for the strength value to be further improved by optimizing the cement replacement and water to binder ratios.
4. The obtained results from XRD and DTG/TGA analysis show an increase in CH consumption of blended cement pastes with curing time. The percentage of fixed lime reaches a maximum of 20.88% after 90 days of curing, suggesting pozzolanic activity equivalent to fly ash.
5. The blended cement pastes with UGCS show compact microstructures with eroded CH, amorphous C–S–H gel, AFt, unreacted UGCS particles, and pores in the cementitious matrix. EDS analysis shows higher Fe/Si ratio and lower Ca/Si of the C–S–H gel in the blended cement pastes than that in the PLC pastes.
